# 3D Multiple-Antenna Channel Modeling and Propagation Characteristics Analysis for Mobile Internet of Things

**DOI:** 10.3390/s21030989

**Published:** 2021-02-02

**Authors:** Wenbo Zeng, Yigang He, Bing Li, Shudong Wang

**Affiliations:** 1 School of Electrical and Automation Engineering, Hefei University of Technology, Hefei 230009, China; zengwenbo0513@163.com (W.Z.); libinghnu@hfut.edu.cn (B.L.); wangshudong92@163.com (S.W.); 2 School of Electrical Engineering, Wuhan University, Wuhan 430072, China

**Keywords:** three-dimensional channel model, multiple-antenna systems, mobile IoT, simulation model, statistical channel characteristics

## Abstract

The demand for optimization design and performance evaluation of wireless communication links in a mobile Internet of Things (IoT) motivates the exploitation of realistic and tractable channel models. In this paper, we develop a novel three-dimensional (3D) multiple-antenna channel model to adequately characterize the scattering environment for mobile IoT scenarios. Specifically, taking into consideration both accuracy and mathematical tractability, a 3D double-spheres model and ellipsoid model are introduced to describe the distribution region of the local scatterers and remote scatterers, respectively. Based on the explicit geometry relationships between transmitter, receiver, and scatterers, we derive the complex channel gains by adopting the radio-wave propagation model. Subsequently, the correlation-based approach for theoretical analysis is performed, and the detailed impacts with respect to the antenna deployment, scatterer distribution, and scatterer density on the vital statistical properties are investigated. Numerical simulation results have shown that the statistical channel characteristics in the developed simulation model nicely match those of the corresponding theoretical results, which demonstrates the utility of our model.

## 1. Introduction

The Internet of Things (IoT) connects a multitude of dissimilar sensors and devices with the Internet through various communication links in a robust and efficient manner to support complex and ubiquitous interactions between physical objects [1]. Such an emerging trend has a steady and sustained penetration into various domains, including industries, intelligent transportation systems, healthcare, smart cities, smart space, and smart grids [2,3,4,5,6,7]. In recent years, mobile and wireless communications have become important enabling technologies to allow the growth of the IoT vision [8]. Typical examples of this are wireless sensor networks (WSNs) and wireless sensor actor networks (WSANs). As essential integral parts of the IoT paradigm, WSNs and WSANs consist of a collection of sensor nodes connected through wireless channels and provide digital interfaces to real-world things [9]. Accompanied by technologies in the information field like big data and blockchain, these large-scale data collected from WSNs can be tapped for potential value for service consumers [10,11]. Moreover, the revolutionary technologies in fifth (and beyond) generation (5G) systems like massive multi-input multi-output (MIMO) are expected to provide high spectral efficiencies and high data rates to satisfy the enormous traffic demands of heterogeneous and scattered communicating units [12,13].

In general, the wireless signal is particularly vulnerable to multipath fading effects as the result of reflection, diffraction, and scattering phenomena. Accordingly, the communication system performance is strictly restricted by the underlying propagation characteristics. In particular, the spatial–temporal correlation properties that result from the dense antenna array or lack of rich scattering are capable of degrading the multiple-antenna system’s performance significantly [14]. The diversity and complexity of the propagation channel resulting from the multi-mobility and fast-changing mobile IoT scenarios make conducting field trials costly and time-consuming, which poses a crucial challenge to the thorough investigation of actual propagation characteristics. Fortunately, channel modeling provides a repeatable and cost-effective way to reproduce the desired channel characteristics by abstracting scene features [15]. Therefore, the development of accurate and efficient fading models capturing key channel characteristics is an indispensable prerequisite for the design optimization and performance evaluation of the mobile IoT communication systems.

Extensive studies in terms of the channel models have been carried out for classical cellular systems. Nevertheless, the direct extension of these models to mobile IoT is infeasible due to the distinct scattering environment where both the transmitter and receiver are in motion and surrounded by scatterers. Broadly speaking, the channel modeling approach can be divided into the following three categories: the correlation-based stochastic model (CBSM) [16], the geometry-based deterministic model (GBDM) [17], and the geometry-based stochastic model (GBSM). Although the CBSM is of low implementation complexity, this oversimplified approach without explicitly accounting for wave propagation usually compromises the precision. Conversely, the GBDM prescribes the reflecting scattering environments in an entirely fixed and exhaustive manner. Nevertheless, the extremely detailed and highly complex description of site-specific environments consequently undermines the generalization capability of this approach to mobile IoT scenarios. It has been demonstrated in the literature that the GBSM has the merits of flexibility and mathematical tractability, and has been applied to theoretical analysis in various high-mobility multiple-antenna scenarios, such as vehicle-to-vehicle [18], high-speed trains [19], and unmanned aerial vehicles [20].

The GBSM considers that the waves experience multipath scattered by the surrounding environments and assumes that the distribution range of the effective scatterers is of regular or irregular geometry. In previous publications, the widely used regular-shaped GBSM includes the double-rings model [21], elliptical model [22], and T-junction model [23]. In [24], the double-rings model was first proposed for isotropic scattering single-input single-output (SISO) Rayleigh fading channels. Building on this, Pätzold considered the von Mises distribution as the scatterer distribution and accordingly extended the applicability of the double-rings model in non-isotropic scattering [25]. However, only double-bounced (DB) rays are taken into account in the models proposed in [24,25], and this setting seems to be inappropriate, particularly for the IoT environment with low-density scatterers, where line-of-sight (LoS) and single-bounced (SB) rays constitute more prominent components. Besides, due to the thoughtless neglect of multiple-bounced links, the elliptical model is short of the describing ability for mobile IoT scenarios with relatively high scatterer density. Scatterer density is an important feature that reflects the mobile IoT communication environment, which has been demonstrated to have an effect on the channel performances, especially on the capacity and correlation properties [26,27]. Few channel models have been devoted to investigating the impact of scatterer density on channel statistics, which motivates more detailed studies. In addition, most previously mentioned models based on the GBSM imposed the acquiescent constraint that transmitters, receivers, and scatterers are located on the same layer and assumed the waves propagate only in the two-dimensional (2D) space. However, in the actual propagation process, the assumption of 2D is extremely insufficient. Research results, as well as field measurements, have shown that there exists a large capacity gap as predicted by three-dimensional (3D) and 2D models, and the gap of correlation grows quadratically due to a slight elevation angle spread [28,29,30]. This highlights the importance of an accurate 3D channel model when evaluating multiple-antennas systems.

Motivated by the above background and gaps of current research, in this paper, a novel 3D multiple-antenna channel model based on the GBSM approach is proposed for mobile IoT communication scenarios. The proposed model invokes the geometrical 3D double-spheres model and ellipsoid model, which is sufficiently generic and adapted to various realistic IoT environmental conditions featuring both local scatterers and remote scatterers by adjusting the corresponding model parameters. Subsequently, according to the exact geometrical relationship among the azimuth angle of departure (AAoD), the azimuth angle of arrival (AAoA), the elevation angle of departure (EAoD), and elevation angle of arrival (EAoA), we derive the critical channel correlation characteristics of the proposed model and investigate the impact of the antenna deployment, scatterer distribution state, and scatterer density on these characteristics. In addition, the corresponding simulation model is presented by leveraging efficient parameter calculation methods. Our study extends the research of channel modeling and provides insights for the design and deployment of mobile IoT communication systems.

The remainder of this paper is organized as follows. In Section 2, we provide the 3D channel model for mobile IoT wireless communication systems. Herein, we derive complex channel gains and determine the distribution of effective scatterers in detail. In Section 3, channel statistical properties are derived, and the simulation parameter calculation method is proposed. Section 4 presents the numerical simulation results. Finally, the conclusions are shown in Section 5.

## 2. Proposed 3D Channel Model

### 2.1. Description of Theoretical Model

A typical wireless communication scenario for mobile IoT environments is considered, where the mobile transmitter (MT) and mobile receiver (MR) are in the motion state. *v_T_* and *v_R_* are the mobile velocities of MT and MR, respectively, with the mobile directions *γ_T_* and *γ_R_*. It is assumed that the MT and MR are equipped with *M_T_* and *M_R_* uniform linear array (ULA) antennas with omnidirectional patterns (i.e., the antenna patterns can be normalized to 1). The antenna elements are spaced with separation *δ_T_* and *δ_R_*. The *p*-th (*p* ∈ {1, 2, …, *M_T_*}) antenna of MT and *q*-th (*q* ∈ {1, 2, …, *M_R_*}) antenna of MR are denoted as *T_q_* and *R_q_*, respectively. Moreover, *O_T_* and *O_R_* denote the antenna center of the MT and MR, respectively. Note that the proposed model can be generalized to other kinds of antenna arrays, such as circular or spherical multielement antenna arrays.

**Figure 1:**
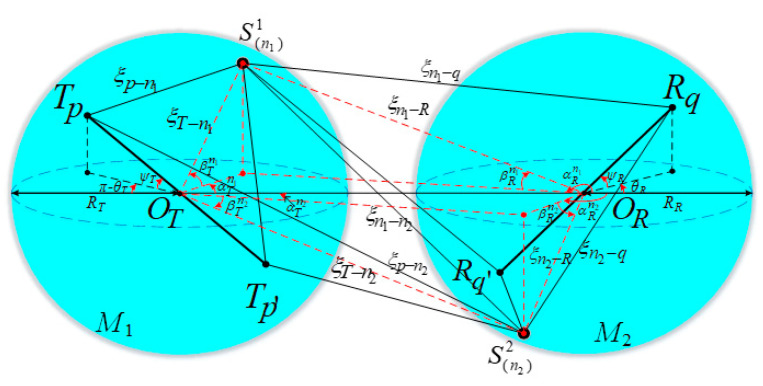
Proposed 3D double-spheres model capturing local scatterer distribution for mobile Internet of Things (IoT) scenarios.

**Figure 2:**
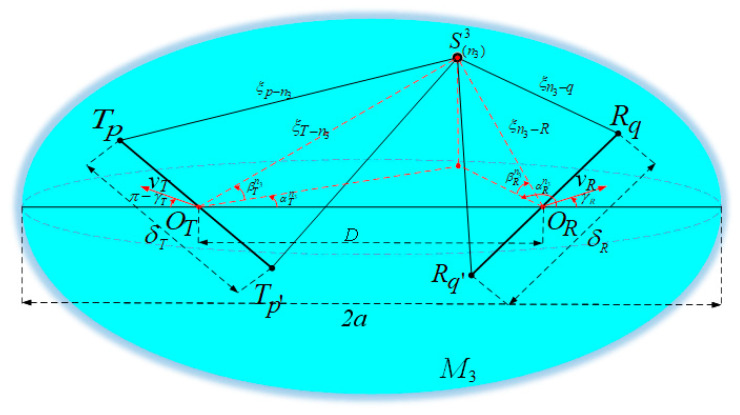
Proposed 3D ellipsoid model capturing remote scatterer distribution for mobile IoT scenarios.

The wave propagation environment is characterized by 3D effective scattering with LoS and non-line-of-sight (NLoS) components, and the NLoS components consist of SB rays and DB rays. In the proposed 3D multiple-antenna regular-shaped geometry-based stochastic model, the distribution region of the local scatterers is modeled by the double-spheres model, as illustrated in Figure 1. Likewise, the distribution of the remote scatterers is modeled by the ellipsoid model, as shown in Figure 2.

The distance between *O_T_* and *O_R_* is denoted as *D*. Here, the single-sphere model with the center *O_T_* and radius *R_T_* is represented as *M*_1_, and the single-sphere model with center *O_R_* and radius *R_R_* is represented as *M*_2_. Besides, the ellipsoid model is represented as *M*_3_ whose focal points are *O_T_* and *O_R_*. The ellipsoid’s semi-length on the major axis is *a*. It is assumed that there exists *N*_1_ effective scatterers located on *M*_1_, and the *n*_1_-th scatterer (*n*_1_ = 1, 2, …, *N*_1_) on *M*_1_ is represented by symbol $S_{\left(n1\right)}^{1}$. *N*_2_ effective scatterers are located on *M*_2_, and the *n*_2_-th scatterer (*n*_2_ = 1, 2, …, *N*_2_) on *M*_2_ is represented by symbol $S_{\left(n2\right)}^{2}$. Similarly, it is assumed that there exists *N*_3_ effective scatterers located on *M*_3_, and the *n*_3_-th scatterer (*n*_3_ = 1, 2, …, *N*_3_) on *M*_3_ is represented by symbol $S_{\left(n3\right)}^{3}$. For NLoS rays, the waves from the MT antenna elements impinge on the scatterers located on *M*_1_, *M*_2_, or *M*_3_ before they arrive at the MR antenna elements, such as SB ray *T_q_* − $S_{\left(n3\right)}^{3}$ − *R_q_* and DB ray *T_q_* − $S_{\left(n1\right)}^{1}$ − $S_{\left(n2\right)}^{2}$ − *R_q_*. The notations and parameters in the model are defined in Table 1. In addition, since the antenna array is generally compact in the multiple-antenna systems, it is reasonably assumed that min {*R_T_*, *R_R_*, *a* − 0.5*D*} >> max {*δ_T_*, *δ_R_*}.

In this proposed 3D channel model, the physical characteristics of a multiple-antenna channel can be described by a complex fading envelope matrix ***H***(*t*). The element in ***H***(*t*) that represents the diffuse component of the transmission link from *T_p_* to *R_q_* is *h_pq_*(*t*). It is assumed that the received complex fading envelope *h_pq_*(*t*) is superimposed by LoS, SB, and DB components, which can be expressed as

(1) $$h_{pq}(t)=\sum_{i=1}^{3}h_{pq}^{\mathrm{SB}i}(t)+h_{pq}^{\mathrm{DB}}(t)+h_{pq}^{\mathrm{LoS}}(t).$$

#### 2.1.1. Line-of-Sight Component

The LoS component can be modeled as

(2) $$h_{pq}^{\mathrm{LoS}}(t)=\sqrt{\frac{K_{pq}\Omega_{pq}}{K_{pq}+1}}e^{j2\pi f_{pq}^{\mathrm{LoS}}t-j\frac{2\pi }{\lambda }\xi_{pq}},$$

where *Ω_pq_* is the total power of the *T_p_* − *R_q_* link, *K_pq_* designates the Ricean factor defined as the ratio of signal power in the dominant component over the scattered power, *λ* is the wavelength and *λ* = *c*/*f_c_*, *c* is the speed of the wave, and *f_c_* is the carrier frequency. $f_{pq}^{\mathrm{LoS}}$ denotes the Doppler frequency of the LoS component due to the motion, which can be calculated as

(3) $$f_{pq}^{\mathrm{LoS}}=f_{Tmax}\cos(\pi -\alpha^{\mathrm{LoS}}-\gamma_{T})\;+f_{Rmax}\cos(\alpha^{\mathrm{LoS}}-\gamma_{R}),$$

where *f_Tmax_* = *v_T_/λ* and *f_Rmax_* = *v_R_/λ* are the maximum Doppler frequencies with respect to the MT and MR, respectively. We also have *α*^LoS^ = *π* because of the assumption that min {*R_T_*, *R_R_*, *a* − 0.5*D*} >> max {*δ_T_*, *δ_R_*}.

Therefore, the LoS path length can be calculated as

(4) $$\xi_{pq}=D-k_{p}\delta_{T}\cos\theta_{T}\cos\psi_{T}\;-\;k_{q}\delta_{R}\cos(\alpha^{\mathrm{LoS}}-\theta_{R})\cos\psi_{R},$$

where *k_p_* = 0.5*M_T_* + 0.5 − *p*, *k_q_* = 0.5*M_R_* + 0.5 − *q*.

#### 2.1.2. Single-Bounced Component

In this model, we assume that there are three single-bounced subcomponents, SB1 from *M*_1_, SB2 from *M*_2_, and SB3 from *M*_3_, which can be modeled as

(5) $$h_{pq}^{\mathrm{SB}1}(t)=\sqrt{\frac{\eta_{\mathrm{SB}1}\Omega_{pq}}{K_{pq}+1}}\underset{N_{1}\to \infty }{\lim}\sum_{n_{1}=1}^{N_{1}}\frac{1}{\sqrt{N_{1}}}e^{j2\pi f_{n_{1}}^{\mathrm{SB}1}t}{}^{-j\frac{2\pi }{\lambda }(\xi_{p-n_{1}}+\xi_{n_{1}-q})},$$

(6) $$h_{pq}^{\mathrm{SB}2}(t)=\sqrt{\frac{\eta_{\mathrm{SB}2}\Omega_{pq}}{K_{pq}+1}}\underset{N_{2}\to \infty }{\lim}\sum_{n_{2}=1}^{N_{2}}\frac{1}{\sqrt{N_{2}}}e^{j2\pi f_{n_{2}}^{\mathrm{SB}2}t-j\frac{2\pi }{\lambda }(\xi_{p-n_{2}}+\xi_{n_{2}-q})},$$

(7) $$h_{pq}^{\mathrm{SB}3}(t)=\sqrt{\frac{\eta_{\mathrm{SB}3}\Omega_{pq}}{K_{pq}+1}}\underset{N_{3}\to \infty }{\lim}\sum_{n_{3}=1}^{N_{3}}\frac{1}{\sqrt{N_{3}}}e^{j2\pi f_{n_{3}}^{\mathrm{SB}3}t-j\frac{2\pi }{\lambda }(\xi_{p-n_{3}}+\xi_{n_{3}-q})},$$

where *η*_SB1_, *η*_SB2_, and *η*_SB3_ are the weights that SB1, SB2, and SB3 rays contribute to the total NLoS power. The Doppler shift of SB*i* components can be expressed as

(8) $$f_{n_{i}}^{\mathrm{SB}i}=f_{Tmax}\cos(\alpha_{T}^{n_{i}}-\gamma_{T})\cos\beta_{T}^{n_{i}}\;+\;f_{Rmax}\cos(\alpha_{R}^{n_{i}}-\gamma_{R})\cos\beta_{R}^{n_{i}}$$

Here, according to the law of cosines and ellipsoid properties, we have

(9) $$\{\begin{array}{c} \xi_{T-n_{1}}=R_{T}\\ \xi_{n_{1}-R}=\sqrt{R_{T}{}^{2}+D^{2}-2R_{T}D\cos\alpha_{T}^{n_{1}}\cos\beta_{T}^{n_{1}}}\\ \xi_{n_{2}-R}=R_{R}\\ \xi_{T-n_{2}}=\sqrt{R_{R}{}^{2}+D^{2}+2R_{R}D\cos\alpha_{R}^{n_{2}}\cos\beta_{R}^{n_{2}}}\\ \xi_{n_{3}-R}=\frac{4a^{2}-D^{2}}{4a+2D\cos\alpha_{R}^{n_{3}}\cos\beta_{R}^{n_{3}}}\\ \xi_{T-n_{3}}=2a-\xi_{n_{3}-R} \end{array}.$$

Based on (9), the path length of SB*i* can be computed as

(10) $$\begin{array}{cc} \xi_{p-n_{1}}+\xi_{n_{1}-q} & \approx R_{T}+\xi_{n_{1}-R}\\ & -k_{p}\delta_{T}\left[\cos\psi_{T}\cos\beta_{T}^{n_{1}}\cos(\alpha_{T}^{n_{1}}-\theta_{T})+\sin\psi_{T}\sin\beta_{T}^{n_{1}}\right]\\ & -k_{q}\delta_{R}\left[\cos\psi_{R}\cos\beta_{R}^{n_{1}}\cos(\alpha_{R}^{n_{1}}-\theta_{R})+\sin\psi_{R}\sin\beta_{R}^{n_{1}}\right] \end{array},$$

(11) $$\begin{array}{cc} \xi_{p-n_{2}}+\xi_{n_{2}-q} & \approx \xi_{T-n_{2}}+R_{R}\\ & -k_{p}\delta_{T}\left[\cos\psi_{T}\cos\beta_{T}^{n_{2}}\cos(\alpha_{T}^{n_{2}}-\theta_{T})+\sin\psi_{T}\sin\beta_{T}^{n_{2}}\right]\\ & -k_{q}\delta_{R}\left[\cos\psi_{R}\cos\beta_{R}^{n_{2}}\cos(\alpha_{R}^{n_{2}}-\theta_{R})+\sin\psi_{R}\sin\beta_{R}^{n_{2}}\right] \end{array},$$

(12) $$\begin{array}{cc} \xi_{p-n_{3}}+\xi_{n_{3}-q} & \approx \xi_{T-n_{3}}+\xi_{n_{3}-R}\\ & -k_{p}\delta_{T}\left[\cos\psi_{T}\cos\beta_{T}^{n_{3}}\cos(\alpha_{T}^{n_{3}}-\theta_{T})+\sin\psi_{T}\sin\beta_{T}^{n_{3}}\right]\\ & -k_{q}\delta_{R}\left[\cos\psi_{R}\cos\beta_{R}^{n_{3}}\cos(\alpha_{R}^{n_{3}}-\theta_{R})+\sin\psi_{R}\sin\beta_{R}^{n_{3}}\right] \end{array}.$$

For SB rays, there exists a correlation among AAoD, AAoA, EAoD, and EAoA. According to the geometrical relationship, the exact relationship can be calculated as follows:

(13) $$\beta_{R}^{n_{1}}=\arcsin\left(\frac{R_{T}\sin\beta_{T}^{n_{1}}}{\xi_{n_{1}-R}}\right),$$

(14) $$\alpha_{R}^{n_{1}}=\arcsin\left(\frac{R_{T}\cos\beta_{T}^{n_{1}}\sin\alpha_{T}^{n_{1}}}{\xi_{n_{1}-R}\cos\beta_{R}^{n_{1}}}\right),$$

(15) $$\beta_{T}^{n_{2}}=\arcsin\left(\frac{R_{R}\sin\beta_{R}^{n_{2}}}{\xi_{T-n_{2}}}\right),$$

(16) $$\alpha_{T}^{n_{2}}=\arcsin\left(\frac{R_{R}\cos\beta_{R}^{n_{2}}\sin\alpha_{R}^{n_{2}}}{\xi_{T-n_{2}}\cos\beta_{T}^{n_{2}}}\right),$$

(17) $$\sin\beta_{T}^{n_{3}}=\frac{\xi_{n_{3}-R}\sin\beta_{T}^{n_{3}}}{\xi_{T-n_{3}}},$$

(18) $$\sin\alpha_{T}^{n_{3}}=\frac{\xi_{n_{3}-R}\cos\beta_{R}^{n_{3}}\sin\alpha_{R}^{n_{3}}}{\xi_{T-n_{3}}\cos\beta_{T}^{n_{3}}},$$

#### 2.1.3. Double-Bounced Component

Similarly, the DB components can be modeled as

(19) $$h_{pq}^{\mathrm{DB}}(t)=\sqrt{\frac{\eta_{\mathrm{DB}}\Omega_{pq}}{K_{pq}+1}}\underset{N_{1},N_{2}\to \infty }{\lim}\sum_{n_{1}}^{N_{1}}\sum_{n_{2}}^{N_{2}}\frac{1}{\sqrt{N_{1}N_{2}}}e^{j2\pi f_{n_{1},n_{2}}^{\mathrm{DB}}t-j\frac{2\pi }{\lambda }(\xi_{p-n_{1}}+\xi_{n_{1}-n_{2}}+\xi_{n_{2}-q})},$$

where *η_DB_* denotes the weight that DB rays contribute to total NLoS power, and all weights satisfy the constraint that *η*_SB1_ + *η*_SB2_ + *η*_SB3_ + *η*_DB_ = 1. The Doppler shift of the DB component can be expressed as

(20) $$f_{n_{1},n_{2}}^{\mathrm{DB}}=f_{Tmax}\cos(\alpha_{T}^{n_{1}}-\gamma_{T})\cos\beta_{T}^{n_{1}}+f_{Rmax}\cos(\alpha_{R}^{n_{2}}-\gamma_{R})\cos\beta_{R}^{n_{2}}$$

The path length for DB components can be computed as

(21) $$\begin{array}{cc} \xi_{p-n_{1}}+\xi_{n_{1}-n_{2}}+\xi_{n_{2}-q} & \approx R_{T}+D+R_{R}\\ & -k_{p}\delta_{T}\left[\cos\psi_{T}\cos\beta_{T}^{n_{1}}\cos(\alpha_{T}^{n_{1}}-\theta_{T})+\sin\psi_{T}\sin\beta_{T}^{n_{1}}\right]\\ & -k_{q}\delta_{R}\left[\cos\psi_{R}\cos\beta_{R}^{n_{2}}\cos(\alpha_{R}^{n_{2}}-\theta_{R})+\sin\psi_{R}\sin\beta_{R}^{n_{2}}\right] \end{array}$$

### 2.2. Distribution of Effective Scatterers

**Figure 3:**
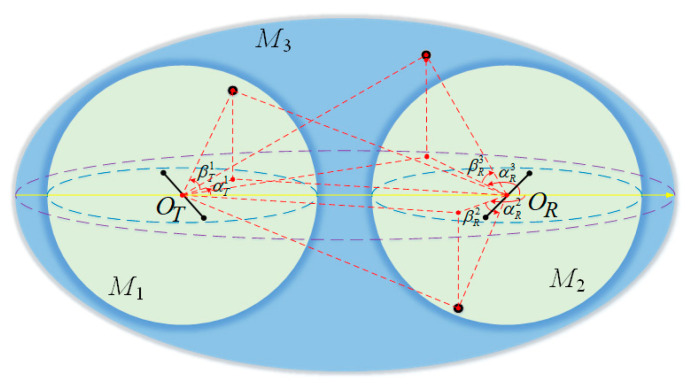
The illustration of the continuous random variables $\alpha_{T}^{1}$, $\alpha_{R}^{2}$, $\alpha_{R}^{3}$, $\beta_{T}^{1}$, $\beta_{R}^{2}$, and $\beta_{R}^{3}$ for the distribution of effective scatterers in the proposed theoretical model.

In the proposed 3D model, the six discrete variables $\alpha_{T}^{n1}$, $\alpha_{R}^{n2}$, $\alpha_{R}^{n3}$, $\beta_{T}^{n1}$, $\beta_{R}^{n2}$, and $\beta_{R}^{n3}$ can determine the location of effective scatterers in *M*_1_, *M*_2_, and *M*_3_. As for the theoretical model, the effective scatterers are assumed to be infinite. Accordingly, the abovementioned discrete random variables can be replaced by continuous random variables $\alpha_{T}^{1}$, $\alpha_{R}^{2}$, $\alpha_{R}^{3}$, $\beta_{T}^{1}$, $\beta_{R}^{2}$, and $\beta_{R}^{3}$, as shown in Figure 3.

We leverage the von Mises distribution and cosine distribution applied in [26] to adaptively depict the probability density function (PDF) of the continuous random variables in the azimuth and elevation planes, respectively, which can be expressed as

(22) $$f_{1}\left(\alpha |\alpha_{0},k\right)=\exp\left[k\cos(\alpha -\alpha_{0})\right]/\left[2\pi I_{0}(k)\right],$$

(23) $$\begin{array}{r} f_{2}\left(\beta |\beta_{0},\beta_{m}\right)=\frac{\pi }{4\beta_{m}}\cos\left(\frac{\pi \left(\beta -\beta_{0}\right)}{2\beta_{m}}\right)\\ \mathrm{where}\;\;\left|\beta -\beta_{0}\right|\leq \beta_{m}\leq \frac{\pi }{2} \end{array},$$

where *α*_0_ and *β*_0_ denote the mean angle of azimuth and elevation, respectively, *β_m_* represents the maximum range of elevation angle deviation from the mean value *β*_0_, and *k* (*k* ≥ 0) denotes the control factor for the distribution concentration relative to *α*_0_. Note that the larger *k* implies more concentrated scatterers. If *k* = 0, the scatterer distribution is isotropic. Contrarily, if *k* = 0, the distribution can characterize the non-isotropic scattering environment. Furthermore, the *I*_0_(·) is the zeroth-order modified Bessel function of the first kind.

In the following derivation, to characterize the scatterers on *M*_1_, *M*_2_, and *M*_3_, the von Mises distribution probability density functions *f*_1_($\alpha_{T}^{1}$), *f*_1_($\alpha_{R}^{2}$), *f*_1_($\alpha_{R}^{3}$) and cosine distribution probability density functions *f*_2_($\beta_{T}^{1}$), *f*_2_($\beta_{R}^{2}$), *f*_2_($\beta_{R}^{2}$) are adopted. Moreover, the parameters {*α*_0_, *β*_0_, *β_m_*, *k*} in (22) and (23) would be replaced by corresponding parameters {$\alpha_{T0}^{1}$, $\beta_{T0}^{1}$, $\beta_{Tm}^{1}$, *k*_1_}, {$\alpha_{R0}^{2}$, $\beta_{R0}^{2}$, $\beta_{Rm}^{2}$, *k*_2_} and {$\alpha_{R0}^{3}$, $\beta_{R0}^{3}$, $\beta_{Rm}^{3}$, *k*_3_}, respectively, which can adaptably characterize a wide variety of mobile IoT scattering environments.

## 3. Channel Statistical Properties and Simulation Model

### 3.1. Sparial–Temporal Correlation Function

The normalized spatial–temporal correlation function (ST-CF) between any two complex fading envelopes is defined as

(24) $$\rho_{pq,p^{\prime }q^{\prime }}(\delta_{T},\delta_{R},\tau )=\frac{E[h_{pq}(t)\cdot h_{p^{\prime }q^{\prime }}{}^{*}(t+\tau )]}{\sqrt{\Omega_{pq}\Omega_{p^{\prime }q^{\prime }}}}.$$

Since the LoS component, SB components, and DB components are independent zero-mean complex Gaussian random processes, the formulation (24) can be represented by the normalized correlation function of each component as

(25) $$\rho_{pq,p^{\prime }q^{\prime }}(\delta_{T},\delta_{R},\tau )=\sum_{i=1}^{3}\rho_{pq,p^{\prime }q^{\prime }}^{\mathrm{SB}i}(\delta_{T},\delta_{R},\tau )+\rho_{pq,p^{\prime }q^{\prime }}^{\mathrm{LoS}}(\delta_{T},\delta_{R},\tau )+\rho_{pq,p^{\prime }q^{\prime }}^{\mathrm{DB}}(\delta_{T},\delta_{R},\tau ).$$

Note that various other existing correlation functions can be obtained from the ST–CF as exceptional cases. For instance, the 2D spatial CF defined as *ρ_pq_*_,*p′q′*_(*δ_T_*, *δ_R_*) equals the ST-CF at *τ* = 0 and the temporal correlation function defined as *ρ_pq_*_,*p′q′*_(*τ*) can be obtained from the ST-CF at *δ_T_* = *δ_R_* = 0. Substituting the corresponding von Mises PDF and cosine PDF into (25), the ST-CF of all components can be derived as follows.

#### 3.1.1. Line-of-Sight Component

(26) $$\rho_{pq,p^{\prime }q^{\prime }}^{\mathrm{LoS}}(\delta_{T},\delta_{R},\tau )=\sqrt{\frac{K_{pq}K_{p^{\prime }q^{\prime }}}{\left(K_{pq}+1)(K_{p^{\prime }q^{\prime }}+1\right)}}e^{\left(\chi_{1}^{\mathrm{LoS}}+\chi_{2}^{\mathrm{LoS}}\right)},$$

where

(27) $$\chi_{1}^{\mathrm{LoS}}=j\frac{2\pi }{\lambda }\left[(p^{\prime }-p)\delta_{T}\cos\theta_{T}\cos\psi_{T}-(q^{\prime }-q)\delta_{R}\cos\theta_{R}\cos\psi_{R}\right],$$

(28) $$\chi_{2}^{\mathrm{LoS}}=j2\pi \tau (f_{Tmax}\cos\gamma_{T}-f_{Rmax}\cos\gamma_{R}),$$

#### 3.1.2. Single-Bounced Component

(29) $$\begin{array}{cc} \rho_{pq,p^{\prime }q^{\prime }}^{\mathrm{SB}i}(\delta_{T},\delta_{R},\tau ) & =\frac{\eta_{\mathrm{SB}i}}{\sqrt{\left(Kpq+1)(Kp^{\prime }q^{\prime }+1\right)}}\underset{N_{i}\to \infty }{\lim}\frac{1}{N_{i}}\sum_{n_{i}=1}^{N_{i}}E\left[e^{\left(\chi_{1}^{\mathrm{SB}i}+\chi_{2}^{\mathrm{SB}i}\right)}\right]\\ & =\frac{\eta_{\mathrm{SB}i}}{\sqrt{\left(Kpq+1)(Kp^{\prime }q^{\prime }+1\right)}}\int_{-\pi }^{\pi }\int_{-\pi }^{\pi }e^{\left(\chi_{1}^{\mathrm{SB}i}+\chi_{2}^{\mathrm{SB}i}\right)}f_{1}(\alpha_{T(R)}^{i})f_{2}(\beta_{T(R)}^{i})d\alpha_{T(R)}^{i}d\beta_{T(R)}^{i} \end{array},$$

where

(30) $$\chi_{1}^{\mathrm{SB}i}=j\frac{2\pi }{\lambda }\left[\begin{array}{l} (p^{\prime }-p)\delta_{T}\cos\psi_{T}\cos\beta_{T}^{i}\cos\left(\alpha_{T}^{i}-\theta_{T}\right)+(p^{\prime }-p)\delta_{T}\sin\psi_{T}\sin\beta_{T}^{i}\\ +(q^{\prime }-q)\delta_{R}\cos\psi_{R}\cos\beta_{R}^{i}\cos\left(\alpha_{R}^{i}-\theta_{R}\right)+(q^{\prime }-q)\delta_{R}\sin\psi_{R}\sin\beta_{R}^{i} \end{array}\right],$$

(31) $$\chi_{2}^{\mathrm{SB}i}=-j2\pi \tau \left[f_{Tmax}\cos\left(\alpha_{T}^{i}-\gamma_{T}\right)\cos\beta_{T}^{i}+f_{Rmax}\cos\left(\alpha_{R}^{i}-\gamma_{R}\right)\cos\beta_{R}^{i}\right],$$

where E[·] is the expectation operator.

It is notable that the integral variables in the ST-CF of SB1 components are $\alpha_{T}^{1}$ and $\beta_{T}^{1}$, while those in SB2 components are $\alpha_{R}^{2}$ and $\beta_{R}^{2}$, and those in SB3 components are $\alpha_{R}^{3}$ and $\beta_{R}^{3}$. Furthermore, the other variables in (30) and (31) can be replaced by the corresponding integral variables using the exact geometrical relationship.

#### 3.1.3. Double-Bounced Component

(32) $$\begin{array}{l} \rho_{pq,p^{\prime }q^{\prime }}^{\mathrm{DB}}(\delta_{T},\delta_{R},\tau )=\frac{\eta_{\mathrm{DB}}}{\sqrt{\left(Kpq+1)(Kp^{\prime }q^{\prime }+1\right)}}\underset{N_{1},N_{2}\to \infty }{\lim}\frac{1}{N_{1}N_{2}}\sum_{n_{1}=1}^{N_{1}}\sum_{n_{2}=1}^{N_{2}}E\left[e^{\left(\chi_{1}^{\mathrm{DB}}+\chi_{2}^{\mathrm{DB}}\right)}\right]\\ =\frac{\eta_{\mathrm{DB}}}{\sqrt{\left(Kpq+1)(Kp^{\prime }q^{\prime }+1\right)}}\;\int_{-\pi }^{\pi }\int_{-\pi }^{\pi }\int_{-\pi }^{\pi }\int_{-\pi }^{\pi }\left[e^{\left(\chi_{1}^{\mathrm{DB}}+\chi_{2}^{\mathrm{DB}}\right)}f(\alpha_{T}^{1})f(\beta_{T}^{1})f(\alpha_{R}^{2})f(\beta_{R}^{2})\right]\;d\alpha_{T}^{1}d\beta_{T}^{1}d\alpha_{R}^{2}d\beta_{R}^{2} \end{array},$$

where

(33) $$\chi_{1}^{\mathrm{DB}}=j\frac{2\pi }{\lambda }\left[\begin{array}{l} (p^{\prime }-p)\delta_{T}\cos\psi_{T}\cos\beta_{T}^{1}\cos\left(\alpha_{T}^{1}-\theta_{T}\right)+(p^{\prime }-p)\delta_{T}\sin\psi_{T}\sin\beta_{T}^{1}\\ +(q^{\prime }-q)\delta_{R}\cos\psi_{R}\cos\beta_{R}^{2}\cos\left(\alpha_{R}^{2}-\theta_{R}\right)+(q^{\prime }-q)\delta_{R}\sin\psi_{R}\sin\beta_{R}^{2} \end{array}\right],$$

(34) $$\chi_{2}^{\mathrm{DB}}=-j2\pi \tau \left[f_{Tmax}\cos(\alpha_{T}^{1}-\gamma_{T})\cos\beta_{T}^{1}+f_{Rmax}\cos(\alpha_{R}^{2}-\gamma_{R})\cos\beta_{R}^{2}\right].$$

Using the Fourier transform, the corresponding Doppler power spectral density (Doppler PSD) can be obtained from the ST-CF as

(35) $$\begin{array}{cc} S_{pq,p^{\prime }q^{\prime }}(\delta_{T},\delta_{R},v) & =F_{T}\left\{\rho_{pq,p^{\prime }q^{\prime }}(\delta_{T},\delta_{R},\tau )\right\}\\ & =F_{T}\left\{\rho_{{}_{pq,p^{\prime }q^{\prime }}}^{\mathrm{LoS}}(\delta_{T},\delta_{R},\tau )\right\}+\sum_{i=1}^{3}F_{T}\left\{\rho_{{}_{pq,p^{\prime }q^{\prime }}}^{\mathrm{SB}i}(\delta_{T},\delta_{R},\tau )\right\}+F_{T}\left\{\rho_{{}_{pq,p^{\prime }q^{\prime }}}^{\mathrm{DB}}(\delta_{T},\delta_{R},\tau )\right\} \end{array},$$

where the $F_{T}\left[\cdot \right]$ denotes the Fourier transform.

### 3.2. Simulation Model

The 3D theoretical channel model described in Section 2 is grounded on the assumption that the number of effective scatterers is infinite, which is non-realizable in the actual mobile IoT communication environments. To develop a corresponding simulation model through the theoretical model, it is necessary to determine the unknown simulation model parameters, i.e., discrete $\alpha_{T}^{n1}$, $\alpha_{R}^{n2}$, $\alpha_{R}^{n3}$, $\beta_{T}^{n1}$, $\beta_{R}^{n2}$, and $\beta_{R}^{n3}$. The efficient simulation parameter calculation method leveraged in this work for AAoA (AAoD) is expressed as

(36) $$\int_{\alpha_{T\left(R\right)0}^{i}-\pi }^{\alpha_{T\left(R\right)}^{n_{i}}}f_{1}\left(\alpha_{T\left(R\right)}^{i}|\alpha_{T\left(R\right)0}^{i},k_{i}\right)d\alpha_{T\left(R\right)}^{i}=\frac{n_{i}-1/4}{N_{i}}.$$

Similarly, the simulation parameter calculation method for EAoA (EAoD) is

(37) $$\beta_{T\left(R\right)}^{n_{i}}=\frac{2\beta_{T\left(R\right)m}^{i}}{\pi }\arcsin\left(\frac{2n_{i}-1}{N_{i}}-1\right)$$

## 4. Numerical Results and Analysis

In this section, the statistical propagation properties of the proposed 3D multi-antenna channel model and evaluation of the simulation model are investigated in detail using numerical results. The simulation platform is built in MATLAB 2019. Some fixed variables are adopted as the following: *f_c_* = 5.9 GHz, *D* = 300 m, *a* = 200 m, *R_T_* = *R_R_* = 5 m, *f_Tmax_* = *f_Rmax_* = *f_max_* = 90.86 Hz, *γ_T_* = *γ_R_* = 0, $\beta_{T0}^{1}$ = $\beta_{R0}^{2}$ = $\beta_{R0}^{3}$ = 0. To simplify the illustration, we assume the same antenna spacing and antenna array elevation angle in the MT and MR, i.e., *δ_T_* = *δ_R_* = *δ*, *ψ_R_* = *ψ_T_* = *ψ*.

### 4.1. Spatial Correlation

#### 4.1.1. Isotropic Scattering Scenarios

When the communications between the MT and MR are in isotropic scattering scenarios, we obtain *k*_1_ = *k*_2_ = *k*_3_ = 0.

**Figure 4:**
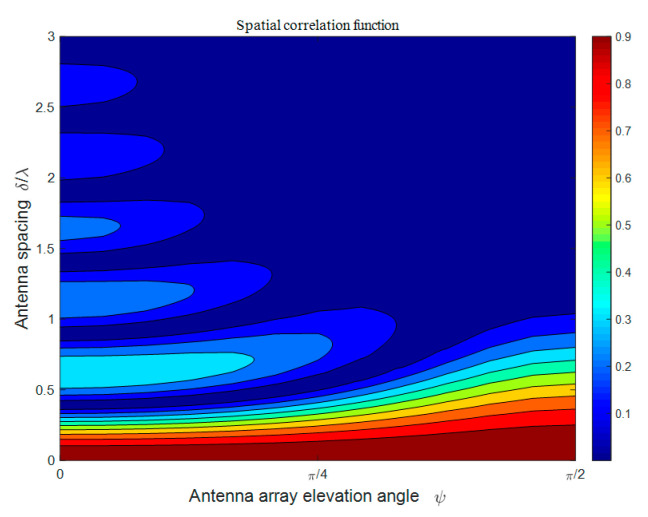
Analytical results of the spatial correlation function (CF) *ρ_pq_*_,*p′q′*_(*δ_T_*, *δ_R_*) of the proposed model in an isotropic scattering environment for different antenna spacings *δ*/*λ* and different antenna array elevation angles *ψ*.

Figure 4 shows the spatial CF of the proposed model in terms of different antenna spacings *δ/λ* and antenna array elevation angles *ψ*. We assume that the scenario is the NLoS condition, i.e., *K_pq_* = 0, and other model parameters are set as: *θ_T_* = *θ_R_* = 0, $\beta_{Tm}^{1}$ = $\beta_{Rm}^{2}$ = $\beta_{Rm}^{3}$ = *π*/6, *η*_SB1_ = *η*_SB2_ =*η*_SB3_ =*η*_DB_ = 0.25. Apparently, in isotropic scattering scenarios, the spatial correlations exhibit an oscillating decrease as the antenna spacing *δ/λ* in the MT/MR increases. This is because the shorter spatial distance of arrays would bring about a more similar channel response of antenna elements, which acts in agreement with the measurements in [31]. In addition, when the antenna spacing *δ/λ* is within a small value, such as *δ/λ* < 1, the spatial correlations tend to be smaller as the antenna array elevation angle *ψ* decreases and could reach the first local minimum faster. Besides, the spatial correlation function oscillates more smoothly in terms of the increasing antenna spacing *δ/λ* in the case of a larger antenna array elevation angle *ψ*. In future deployments of mobile IoT communication, due to the promotion of massive MIMO as well as economic considerations, the antenna array will tend to be miniaturized and compact. Accordingly, adjusting the antenna elevation angle would be taken into consideration to obtain a richer horizontal space, which leads to increased spatial correlation. Therefore, in the actual antenna layout, it should obtain a reasonable trade-off between horizontal spatial redundancy and channel spatial correlation.

**Figure 5:**
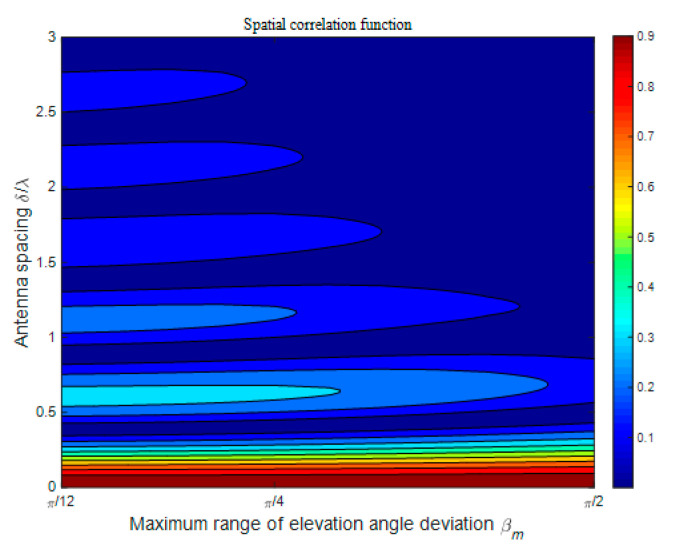
Analytical results of the spatial CF *ρ_pq_*_,*p′q′*_(*δ_T_*, *δ_R_*) of the proposed model in an isotropic scattering environment for different antenna spacings *δ*/*λ* and different maximum ranges of elevation angle deviation *β_M_*.

Figure 5 presents the spatial CF of the proposed model in terms of different antenna spacings *δ/λ* and maximum range of elevation angle deviation *β_M_*. Here, we assume that $\beta_{TM}^{1}$ = $\beta_{RM}^{2}$ = $\beta_{RM}^{3}$ = *β_M_*. Other model parameters are set as: *K_pq_* = 0, *ψ_R_* = *ψ_T_* = *ψ* = 0, *θ_T_* = *θ_R_* = 0, *η*_SB1_ = *η*_SB2_ = *η*_SB3_ = *η*_DB_ = 0.25. From Figure 5, we can observe that the spatial CFs have a similar oscillation frequency but a distinct oscillation amplitude in the cases of different *β_M_*. Overall, the amplitude value decreases with both the increase in antenna spacing *δ/λ* and the increase in the maximum range of elevation angle deviation *β_M_*. Building on these observations, we can conclude that the larger the maximum range of elevation angle deviation *β_M_*, the smaller the spatial correlation characteristics between antenna elements. This conclusion can be explained qualitatively that the expansion of the scatterer distribution range in the vertical dimension means a richer scattering environment, and the different antenna elements have less possibility to be affected by the same range of scatterers. These observations also indicate that 2D wireless channel models would tend to overestimate the channel spatial correlation properties.

**Figure 6:**
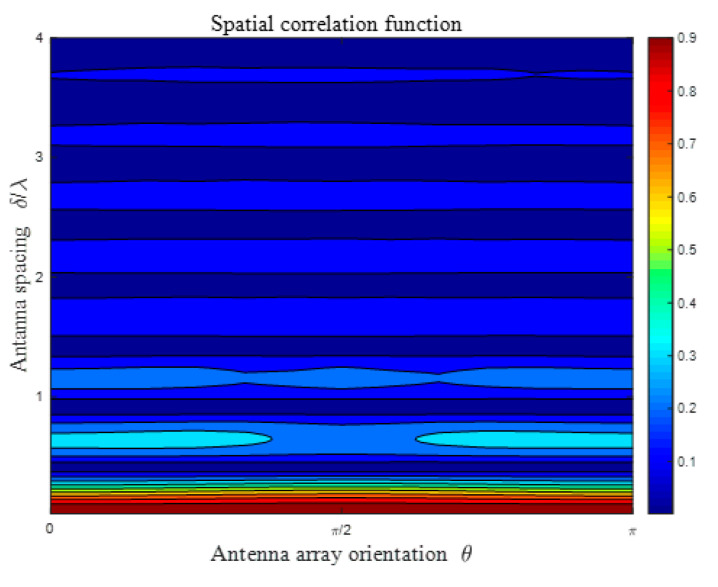
Analytical results of the spatial CF *ρ_pq_*_,*p′q′*_(*δ_T_*, *δ_R_*) of the proposed model in an isotropic scattering environment for different antenna spacings *δ*/*λ* and different antenna array orientation maximum ranges of elevation angle *θ*.

Figure 6 presents the spatial CF of the proposed model in terms of different antenna spacings *δ/λ* and antenna array orientations *θ* = *θ_T_* = *θ_R_*. Other model parameters are set as: *K_pq_* = 0, *ψ_R_* = *ψ_T_* = *ψ* = 0, $\beta_{Tm}^{1}$ = $\beta_{Rm}^{2}$ = $\beta_{Rm}^{3}$ = *π*/6, *η*_SB1_ = *η*_SB2_ = *η*_SB3_ = *η*_DB_ = 0.25. For different values of antenna array orientation *θ*, the resulting spatial correlation functions seem to be approximately indistinguishable. The negligible nuance of these spatial correlation functions results from the geometrical relationship for SB rays, which means that the AAoA and AAoD cannot obey the uniform distribution simultaneously. Hence, we can easily conclude that there exists no correlation between spatial correlation and antenna array orientation under the circumstances of isotropic scattering.

#### 4.1.2. Non-Isotropic Scattering Scenarios

The non-isotropic scattering scenarios can be characterized by setting *k* to a non-zero value.

**Figure 7:**
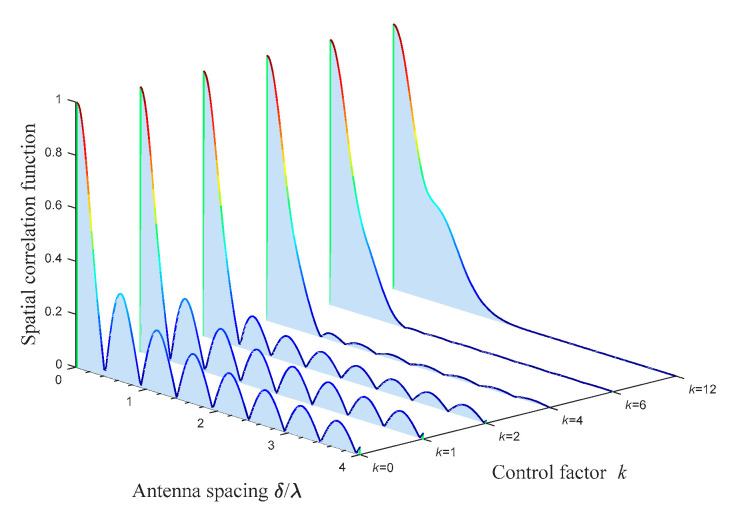
Analytical results of the spatial CF *ρ_pq_*_,*p′q′*_(*δ_T_*, *δ_R_*) of the proposed model for different antenna spacings *δ*/*λ* and different control factors for the distribution concentration *k*.

The impact of the control factor for the distribution concentration *k* of scatterers on the spatial CF is shown in Figure 7. The corresponding parameter settings are: *K_pq_* = 0, *θ_T_* = *θ_R_* = 0, $\beta_{Tm}^{1}$ = $\beta_{Rm}^{2}$ = $\beta_{Rm}^{3}$ = *π*/6, *ψ* = 0, *η*_SB1_ = *η*_SB2_ = *η*_SB3_ = *η*_DB_ = 0.25, $\alpha_{T0}^{1}$ = *π*/4, $\alpha_{R0}^{2}$ = $\alpha_{R0}^{3}$ = 3*π*/4. Comparing the spatial correlation in the cases with different degrees of scatterer concentration, we can find that the spatial correlation increases significantly with the increase in *k* when antenna spacing *δ/λ* is in a small value range. The physical meaning can be understood as when the effective scatterers are more closely distributed, the stronger the influence that the multiple-antenna array elements suffer from effective scatterers in the same area, and the stronger the spatial correlation between antennas. In addition, it can be observed that, compared to the highly concentrated scatterer scene where *k* is large, the spatial correlation in the low scatterer concentration scene declines faster amid more intense fluctuations.

**Figure 8:**
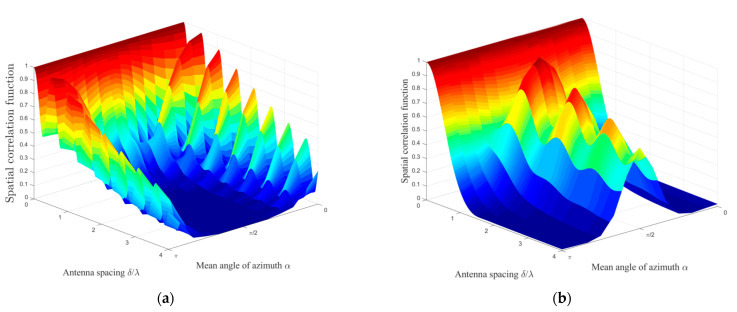
Analytical results of the spatial CF *ρ_pq_*_,*p′q′*_(*δ_T_*, *δ_R_*) of the proposed model in a non-isotropic scattering environment for different antenna spacings *δ*/*λ* and different mean angles *α*_0_ with (**a**) *θ_T_* = *θ_R_* = *0* and (**b**) *θ_T_* = *θ_R_* = *π*/4.

The influence of the parameter *α*_0_ on the spatial correlation characteristics is shown in Figure 8 with (a) *θ_T_* = *θ_R_* = 0 and (b) *θ_T_* = *θ_R_* = *π*/4. The antenna spacing *δ/λ* and $\alpha_{T0}^{1}$ are chosen as the research variables, and we also set the constraint that $\alpha_{R0}^{2}$ = $\alpha_{R0}^{3}$ = *π* − $\alpha_{T0}^{1}$. The other scenario parameters are chosen as: *K_pq_* = 0, *k* = 6, *ψ* = 0, $\beta_{Tm}^{1}$ = $\beta_{Rm}^{2}$ = $\beta_{Rm}^{3}$ = *π*/6, *η*_SB1_ = *η*_SB2_ = *η*_SB3_ = *η*_DB_ = 0.25. Here, we denote *ω* as the angle between the mean of azimuth *α*_0_ and antenna array orientation *θ*, i.e., *ω* = *|α*_0_ − *θ|*. Comparing the distinct spatial correlation properties in Figure 8a,b, we can conclude that the spatial correlation properties are closely related to the angle *ω*. To be specific, when *ω* is within the 0–*π*/4 range, the spatial correlation decreases monotonically with the increase in *ω*. On the contrary, the spatial correlation has a monotonically incremented property if *ω* is in the *π*/4–*π*/2 range. The interesting observation is that the spatial correlation would achieve the global minimum when angle *ω* is a right angle, which would provide certain insights for the design and deployment of the antenna array in mobile IoT communication systems. 

### 4.2. Spatial–Temporal Correlation

**Figure 9:**
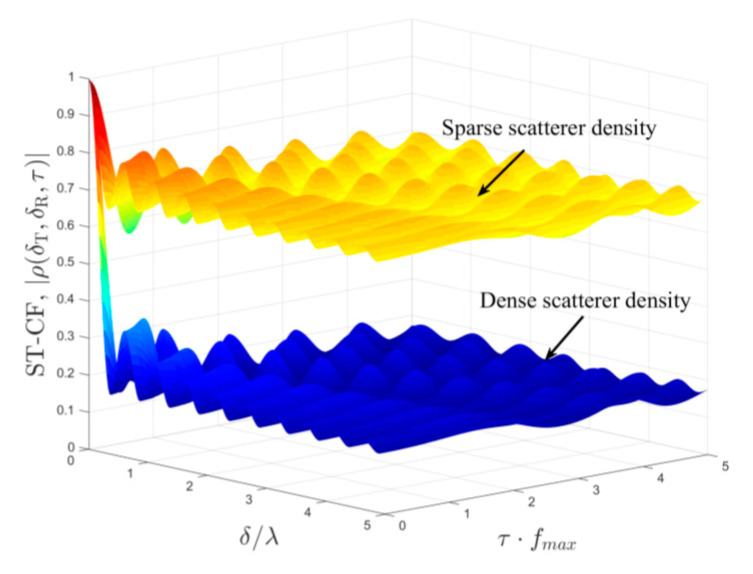
Analytical results of the spatial–temporal correlation function (ST-CF) *ρ_pq_*_,*p′q′*_(*δ_T_*, *δ_R_,τ*) of the proposed model for low scatterer density and high scatterer density.

Scatterer density is an important feature reflecting the communication conditions in mobile IoT wireless transmission scenarios. Herein, we focus our attention on the impact of scatterer density on the spatial–temporal correlation. For a sparse scatterer density, the LoS component bears a significant amount of power. Additionally, SB rays instead of DB rays are more likely to exist, and the local scatterers located in the double-spheres model have a relatively weaker effect on channel propagation than that of remote scatterers. Conversely, for a dense scatterer density, the LoS component is relatively weak, and the DB rays are the primary components of the received signal. Therefore, the mobile IoT scenarios with considerations for scatterer density can be characterized adequately in the proposed channel model by utilizing an appropriate Ricean factor and weights of power contribution. Figure 9 illustrates the spatial–temporal correlation with considerations for scatterer density, where the corresponding model parameters capturing scatterer density features are set as the following: (1) for high scatterer density, *K_pq_* = 0.2, *η_SB1_* = *η_SB2_* = 0.115, *η_SB3_* = 0.055, *η_DB_* = 0.715. (2) For sparse scatterer density, *K_pq_* = 2.186, *η_SB1_* = *η_SB2_* = 0.252, *η_SB3_* = 0.481, *η_DB_* = 0.005. The isotropic scattering is chosen as the mobile IoT communication environment, and other scenario parameters are *ψ* = 0, *θ* = 0, $\beta_{Tm}^{1}$ = $\beta_{Rm}^{2}$ = $\beta_{Rm}^{3}$ = *π*/6, *γ_T_* = *γ_R_* = *π*/2. From Figure 9, we can observe that scatterer density significantly affects the spatial–temporal correlation. Higher scatterer density leads to significantly lower correlation properties thanks to the richer scattering.

### 4.3. Simulation Model

The performance evaluation of the simulation model lies in the better fit of the statistical characteristics of the theoretical model when the scatterer number is limited. Here, the theoretical ST-CF is regarded as the channel characteristic fitting target of the simulation model, and the absolute error is introduced as the appropriate measure for the quality of the approximation between the theoretical model and simulation model, which is defined as

(38) $$e(\delta_{T},\delta_{R},\tau )=\left|\rho (\delta_{T},\delta_{R},\tau )-\tilde{\rho }(\delta_{T},\delta_{R},\tau )\right|,$$

where $\rho (\delta_{T},\delta_{R},\tau )$ and $\tilde{\rho }(\delta_{T},\delta_{R},\tau )$ denote the ST-CF obtained from the theoretical model and simulation model, respectively.

**Figure 10:**
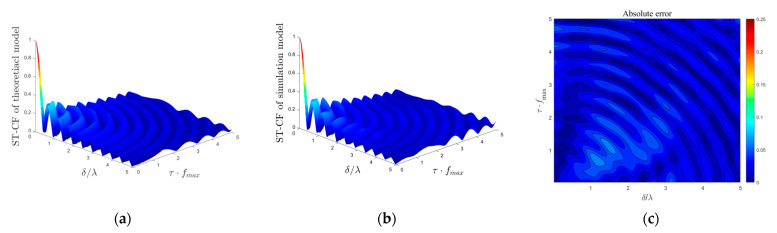
(**a**) ST-CFs of theoretical model in an isotropic scattering environment, (**b**) ST-CFs of simulation model in an isotropic scattering environment, and (**c**) the corresponding absolute error between (**a**,**b**).

**Figure 11:**
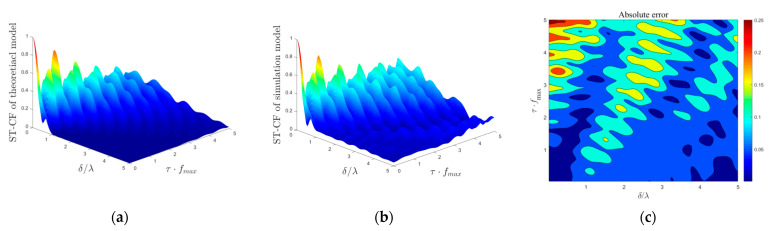
(**a**) ST-CFs of theoretical model in a non-isotropic scattering environment, (**b**) ST-CFs of simulation model in a non-isotropic scattering environment, and (**c**) the corresponding absolute error between (**a**,**b**).

In Figure 10 and Figure 11, we compare the difference in the simulation ST-CF from the desired theoretical ST-CF by adopting the squared error for isotropic scattering scenarios and non-isotropic scattering scenarios, respectively. The number of discrete scatterers in *M*_1_, *M*_2_, and *M*_3_ is selected in the numerical simulation as: *N*_1_ = *N*_2_ = *N*_3_ = 50. The scenario parameters in Figure 9 are: *K_pq_* = 0, *η*_SB1_ = *η*_SB2_ = *η*_SB3_ = *η*_DB_ = 0.25, *k*_1_ = *k*_2_ = *k*_3_ = 0, *ψ* = 0, *θ* = 0, $\beta_{Tm}^{1}$ = $\beta_{Rm}^{2}$ = $\beta_{Rm}^{3}$ = *π*/6, *γ_T_* = *γ_R_* = *π*/2, and those parameters in Figure 10 are: *K_pq_* = 0, *η*_SB1_ = *η*_SB2_ = *η*_SB3_ = *η*_DB_ = 0.25, *k*_1_ = *k*_2_ = *k*_3_ = 6, *ψ* = 0, *θ* = 0, $\beta_{Tm}^{1}$ = $\beta_{Rm}^{2}$ = $\beta_{Rm}^{3}$ = *π*/6, *γ_T_* = *γ_R_* = *π*/2, $\alpha_{T0}^{1}$ = *π*/2, $\alpha_{R0}^{2}$ = $\alpha_{R0}^{3}$ = 3*π*/2. The results obtained in Figure 10 and Figure 11 show that the ST-CFs of the mathematical theoretical model and simulation model match very well, demonstrating the excellent validity of our simulation model. In addition, the fitting effect of the spatial–temporal correlation characteristics of the isotropic scattering environment is better than that of the non-isotropic scattering environment.

## 5. Conclusions

In this paper, we have developed and studied a novel 3D multiple-antenna theoretical channel model and a corresponding simulation channel model for mobile IoT environments. In the proposed model, the double-spheres model and ellipsoid model are leveraged to characterize the efficient local scattering and remote efficient scattering region, respectively. Flexible parameters invest the model with the ability to sufficiently adapt to various mobile IoT scenarios, which provides the model with the capacity to investigate the impact of the scatterer distribution state, antenna deployment, and scatterer density. We derive the ST-CF and corresponding spatial Doppler power spectral density for both isotropic and non-isotropic scattering scenarios. It has been demonstrated that the scatterer distribution concentration would influence oscillation trend of spatial correlation and the higher scatterer density leads to significantly lower correlation properties. In addition, the angle between the mean of the azimuth and antenna array orientation is shown to be a critical factor to determine the spatial correlation in a non-isotropic environment. Those useful conclusions observed by numerical simulations can provide enlightenment on the optimized design of mobile IoT communication systems. Finally, excellent agreement is achieved between the theoretical model and simulation model, which validates the utility of our analysis and derivations.

## Figures and Tables

**Table 1:** The definition of notations and parameters in proposed model.

Notations or Parameter	Definition
*M_T_* (*M_R_*)	The number of antenna of MT (MR)
*T_q_* (*R_q_*)	The *p*-th (*q*-th) antenna of MT (MR)
*O_T_* (*O_R_*)	The antenna center of MT and MR
*M*_1_ (*M*_2_)	The single-sphere around MT (MR)
*M*_3_	The ellipsoid model
*N_i_*	The number of effective scatterers on the model *M_i_*
$S_{\left(ni\right)}^{i}$	The *n_i_*-th scatterer on the model *M_i_*
*R_T_* (*R_R_*)	radius of *M*_1_ (*M*_2_)
*a*, *D*	semi-major axis and focal length of *M*_3_
*δ_T_* (*δ_R_*)	antenna element spacing at MT (MR)
*θ_T_* (*θ_R_*)	antenna array orientation of MT (MR)
*ψ_T_* (*ψ_R_*)	antenna array elevation angle of MT (MR)
*v_T_* (*v_R_*)	mobile velocities of MT (MR)
*γ_T_* (*γ_R_*)	mobile directions of MT (MR)
*α^LoS^*	AAoA of LoS path
$\alpha_{T}^{ni}$, $\alpha_{R}^{ni}$	AAoD and AAoA impinged on the effective $S_{\left(ni\right)}^{i}$,
$\beta_{T}^{ni}$, $\beta_{R}^{ni}$	EAoD and EAoA impinged on the effective $S_{\left(ni\right)}^{i}$
*ξ_pq_*, *ξ_p-ni_*, *ξ_ni-q_*, *ξ_n_*_1-*n*2_, *ξ_T-ni_*, *ξ_ni-R_*	distance of (*T_p_* − *R_q_*), (*T_p_* − $S_{\left(ni\right)}^{i}$), ($S_{\left(ni\right)}^{i}$ − *R_q_*), ($S_{\left(n1\right)}^{1}$ − $S_{\left(n2\right)}^{2}$), (*O_T_* − $S_{\left(ni\right)}^{i}$), ($S_{\left(ni\right)}^{i}$ − *O_R_*)
